# LA-GM-CSF, a Long-Acting Cytokine Mitigates and Prevents H-ARS Mediated Lethality in Mice Exposed to Total Body Gamma Radiation

**DOI:** 10.3390/ijms27094147

**Published:** 2026-05-06

**Authors:** Gregory P. Holmes-Hampton, Kaylee Valenzia, Vidya P. Kumar, Venkateshwara Rao Dronamraju, Ashley Woods, Sean B. Joseph, Sanchita P. Ghosh

**Affiliations:** 1 Armed Forces Radiobiology Research Institute, Uniformed Services University of the Health Sciences, Bethesda, MD 20889, USA; 2 Henry M. Jackson Foundation for Advancement of Military Medicine, Bethesda, MD 20817, USA; 3 Calibr, a Division of Scripps Research, La Jolla, CA 92037, USA

**Keywords:** LA-GM-CSF, total body irradiation, radiation countermeasure, survival, hematopoiesis, biomarkers, sex differences, safety

## Abstract

Widespread uses of nuclear materials increase the risk of accidental or intentional radiation exposure, which can result in acute radiation syndrome (ARS). Hematopoietic ARS (H-ARS) occurs at relatively low doses and is potentially lethal without intervention. While several FDA-approved cytokine-based radiomitigators exist, many require repeated dosing, complicating deployment in mass-casualty scenarios. This study evaluated a novel long-acting, murine-reactive granulocyte–macrophage colony-stimulating factor (LA-GM-CSF; mPDM608) as a prophylactic and mitigative countermeasure for H-ARS. Male and female C57BL/6 mice were exposed to lethal or sublethal total body irradiation (TBI) and treated with LA-GM-CSF using single- or multi-dose regimens administered before or after TBI. Safety, 30-day survival, hematologic recovery, bone marrow cellularity, serum GM-CSF pharmacokinetics, endothelial injury markers, and cytokine profiles were assessed using standard hematology, histopathology, ELISA, and multiplex assays. LA-GM-CSF was well tolerated at doses up to 30 mg/kg. Single or limited dosing conferred significant survival benefits compared with vehicle controls, with optimal efficacy observed at lower doses (3 mg/kg). Post-TBI administration as a single dose 24 h after exposure markedly improved survival in both sexes, with stronger hematopoietic recovery in males. LA-GM-CSF accelerated recovery of neutrophils, red blood cells, platelets, hematocrit, and sternal megakaryocytes, prolonged circulating GM-CSF levels, and favorably modulated endothelial injury markers and select cytokines. LA-GM-CSF demonstrates strong potential as a next-generation radiation countermeasure, providing robust survival benefit and hematopoietic recovery with minimal dosing. The results shown here support further development for H-ARS management under the FDA Animal Rule.

## 1. Introduction

In the modern world, the use of nuclear materials and potential for exposure to radiation is prevalent, including nuclear reactors for isotope and energy production [1], nuclear medicine and radiotherapy [2,3,4], in weapons systems [5], and galactic space radiation [6]. All of these processes lead to the increased likelihood of exposure to nuclear materials, either as a result of accidental or intentional means [7]. Radiation exposure is well documented to result in deleterious symptoms collectively termed acute radiation syndrome (ARS). ARS comprises sub-syndromes which manifest different symptoms depending on the dose of exposure. At low to moderate doses, the hematopoietic system is impacted, resulting in H-ARS [8,9], at higher doses, the gastrointestinal tract is impacted, resulting in GI-ARS, and at the highest doses, the central nervous system is impacted, resulting in CNS-ARS. Despite H-ARS occurring at the lowest doses, it can still be lethal without medical management [10]. The medical management of radiation exposure has been aided by the approval of several mitigators, all studied under the guidance of the FDA Animal Rule.

In the past decade, substantial progress has been made on the development and approval of radiomitigators for the treatment of H-ARS [11,12]. The first approval for H-ARS treatment was the granulocyte colony-stimulating factor (G-CSF, filgrastim) under the trade name Neupogen by Amgen Inc. in March of 2015 [13]. Neupogen increases survival of radiation exposure through the production and maturation of blood cell lineages, particularly neutrophils. This leads to a shortened period of neutropenia, thereby decreasing the period of increased susceptibility to infection. While Neupogen represented a success story in the field of radiation countermeasures, there were also downfalls, particularly the need for daily subcutaneous dosing in patients until the absolute neutrophil counts (ANC) return to healthy levels. The daily dosing is required due to the short half-life of Neupogen, ~3.5 h [14]. Later in the same year, November 2015, a pegylated version of G-CSF, pegfilgrastim, under the trade name Neulasta by Amgen Inc., was approved as a radiomitigator [15]. As a G-CSF analog, pegfilgrastim works by the same mechanism as Neupogen; however, the addition of the polyethylene glycol moiety infers a longer half-life, ~42 h, reducing the dosing requirements to two doses subcutaneously administered 1 week apart [16].

A few years later, in March 2018, granulocyte–macrophage colony-stimulating factor (GM-CSF, sargramostim) under the trade name Leukine was approved [17] for ARS treatment. Leukine also has a short half-life of ~1–2 h [18] and like Neupogen requires daily dosing until threshold ANC’s are met. Unlike G-CSF, GM-CSF does not exhibit cross-reactivity in humans and mice [19]. This presents a unique challenge in the context of the FDA Animal Rule. Generally, Animal Rule approvals include one small animal model (e.g., rodent) and a large animal (e.g., nonhuman primates); however, given that human GM-CSF is not cross-reactive in mice and mouse GM-CSF is not cross-reactive in humans, the use of species-specific protein is required for pre-clinical investigations. Efforts have been made to extend the half-life of GM-CSF, including the production of a Pegylated GM-CSF [20] and the use of protein cross-linked GM-CSF, which is the topic of the current work [21].

The final novel radiation countermeasure, a thrombopoietin mimetic (TPOm) molecule, romiplostim, under the trade name Nplate by Amgen Inc., was approved in January 2021. Nplate features a relatively long half-life of 3.5 days [22], which results in the need for a single dose for the treatment of H-ARS. As a thrombopoietin mimetic, Nplate boosts platelet production in irradiated subjects to reduce the risk of lethality due to bleeding complications. Following the approval of the four parent molecules (Neupogen (G-CSF), Neulasta (Peg-G-CSF), Leukine (GM-CSF), and Nplate (TPOm) the FDA has also approved a further seven biosimilars to Neupogen and Neulasta. These include the four Neulasta biosimilars Udenyca, Stimufend, Ziextenzo, and Fylnetra approved in November 2022, September 2023, February 2024, and April 2025, respectively, and three Neupogen biosimilars Nypozi, Zarxio, and Releuko, approved in June 2024, October 2024, and April 2025, respectively.

In the event of a mass casualty scenario, it is highly anticipated the resources will be limited and infrastructure may be heavily impacted [23,24]. To this end, strategies that involve minimal medical intervention are likely to be preferred. The treatment options that include a single dose (e.g., Nplate) or two doses (e.g., Neulasta) are likely to be preferred over daily dosing, in addition, fewer doses are generally linked to fewer adverse events associated with repeated dosing. However, there is evidence to suggest that GM-CSF may exhibit increased efficacy over G-CSF in instances of delayed administration, a distinct possibility in a mass casualty scenario where shelter-in-place orders may limit prompt intervention [25,26]. In the current study, we investigate the effects of the novel murine reactive LA-GM-CSF compound mPDM608 from the California Institute of Biomedical Research (Calibr), Scripps [21] for increasing survival and providing better clinical outcomes in terms of complete blood cell counts, production of new bone marrow, and altered levels of cytokines in murine subjects exposed to lethal doses of total body irradiation.

## 2. Results

### 2.1. LA-GM-CSF in a 3-Dose Regimen Was Found to Be Safe in C57BL/6 Mice

**Figure 1:**
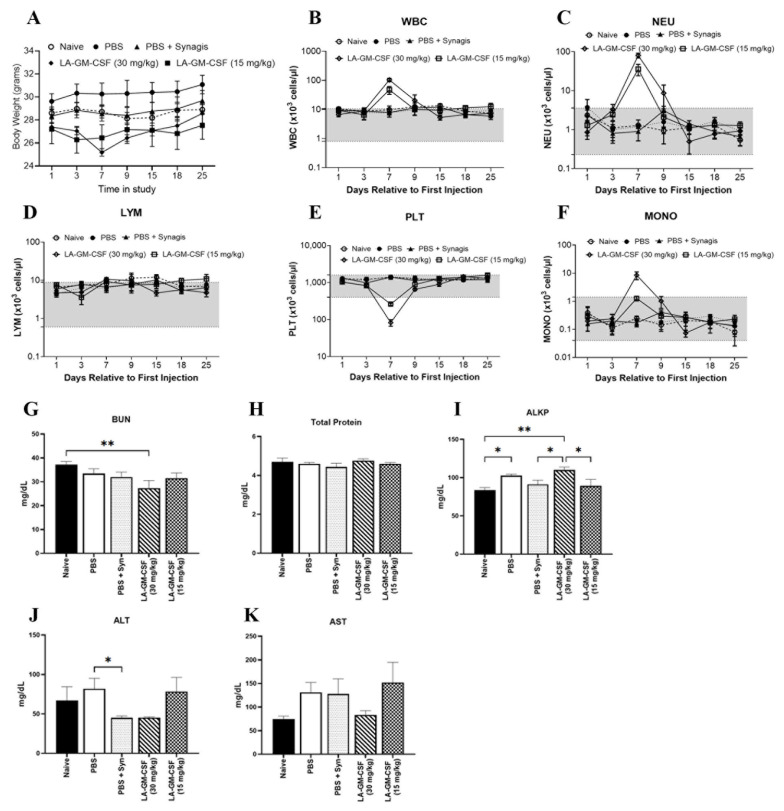
Long-acting cytokine LA-GM-CSF was found to be safe in C57BL/6 mice at doses of 15 and 30 mg/kg (**A**–**K**). Male mice were given LA-GM-CSF (15 or 30 mg/kg) on days 1, 6 and 11. Animals given PBS or PBS with Synagis were used as controls and naïve animals were used as the untreated controls. Body weights recorded (**A**) and complete blood count (CBC) were carried out (**B**–**G**) on days 1, 3, 7, 9, 15, 18 and 25. Serum chemistry parameters (**H**–**K**) for renal and hepatic panels, such as blood urea nitrogen (BUN), alkaline phosphatase (ALKP) and aminotransferases (ALT) and Aspartate aminotransferase (AST), as well as total protein, were measured from serum collected on day 25. Gray area indicates normal range of CBC (**B**–**G**) and serum chemistry (**H**–**K**) for mice. Data is represented as mean ± SEM and statistical significance (indicated as ** (*p* ≤ 0.001), and * (*p* = 0.05)) is calculated by Tukey’s multiple comparisons test. n = 5 animals/group.

Prior to survival efficacy studies, LA-GM-CSF was tested for safety and tolerability in C57BL/6 male mice and was found to be safe at 15 mg/kg with no abnormal clinical signs of toxicity. A 14-day (following the last dose) safety study was performed with LA-GM-CSF; mice were given a dose of either 15 mg/kg/dose or 30 mg/kg/dose LA-GM-CSF or its vehicle control Synagis in PBS on days 1, 6 and 11. The body weights recorded on days 1, 3, 7, 9, 15, 18, and 25 (Figure 1A) showed no significant difference in the groups on all days except on day 7, LA-GM-CSF at a dose of 30 mg/kg showed decreased (*p* = 0.027) weight (25.2 ± 0.3 g) compared to untreated naïve animals (28.7 ± 0.6 g). Peripheral blood counts were also measured on the same days as the body weights. Changes in the counts of white blood cells (WBC), neutrophils (NEU), platelets (PLT) and monocytes (MONO) were observed on day 7, which was a day after the second administration of the LA-GM-CSF. The levels were back to normal by day 9 (by the next reading) (Figure 1B–G). The changes seen on the other days were within the normal range for mice (gray area in Figure 1B–G). On day 7, WBC levels were elevated (*p* < 0.001) in LA-GM-CSF groups (103.92 ± 6.09 × 10^3^ cells/µL and 47.23 ± 6.37 × 10^3^ cells/µL) compared to naïve mice (9.79 ± 1.51 × 10^3^ cells/µL). As for the neutrophils, their levels too were elevated (*p* < 0.001) in LA-GM-CSF groups on day 7 (80.45 ± 5.00 × 10^3^ cells/µL and 35.87 ± 5.21 × 10^3^ cells/µL) compared to naïve mice (1.29 ± 0.217 × 10^3^ cells/µL). Lower trend was seen on day 9 in the 30 mg/kg group, yet higher (*p* = 0.031) compared to naïve mice (0.94 ± 0.077 × 10^3^ cells/µL). Lower platelet levels were observed in the higher dose (30 mg/kg) of LA-GM-CSF on day 7, whereas higher levels of monocytes (*p* = 0.01, 8.41 ± 1.15 × 10^3^ cells/µL) compared to the naïve group (0.25 ± 0.02 × 10^3^ cells/µL). Lymphocyte levels stayed in the normal range for all treated groups. 

Serum chemistry results (Figure 1H–K) on day 25 showed lower Blood Urea Nitrogen (BUN) levels (27.33 ± 3.18 mg/dL) in only the 30 mg/kg LA-GM-CSF-treated group compared to the untreated naïve group (37.25 ± 1.31 mg/dL). Higher levels of alkaline phosphatase (ALKP) (110 ± 3.79 mg/dL) were seen in animals given only 30 mg/kg of LA-GM-CSF compared to naïve animals (83.5 ± 3.59 mg/dL). Alanine transferase (ALT) was lower in animals given a higher dose of LA-GM-CSF, as well as those that received Synagis (45.33 ± 0.88 mg/dL and 45.2 ± 2.24 mg/dL, respectively) compared to naïve animals (67.25 ± 17.2 mg/dL) (Figure 1E). Aspartate aminotransferase (AST) levels were higher but not statistically significant in animals given only 30 mg/kg LA-GM-CSF (83.33 ± 8.84 mg/dL) compared to naïve animals (74.7 ± 6.17 mg/dL).

One of five animals that received LA-GM-CSF (15 mg/kg) died a day after the second administration, although a pathology report (data not included) of the animal could not establish a definitive cause of death. The probable condition could have been murine urologic syndrome, a known genetic condition in this strain of animals [27]. There was no evidence of toxicity as the cause of death attributable to LA-GM-CSF.

### 2.2. Significant Survival Benefit with Single or Multiple Doses of LA-GM-CSF Administered Either Prior to or Post-TBI

**Figure 2:**
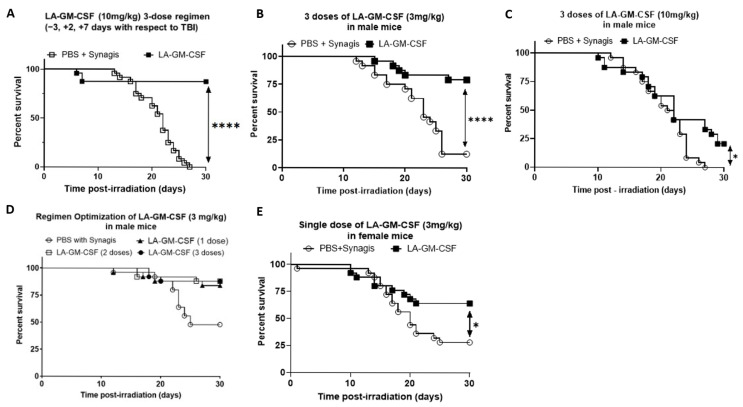
Significant survival benefit by LA-GM-CSF administration as 1-dose, 2-dose, and 3-dose regimens in male and female mice exposed prior to or post-TBI. Kaplan–Meier curves for 30-day survival for male mice given LA-GM-CSF on −3, +2, +7 days with respect to TBI at 10 mg/kg (**A**); on days 1, 6 and 11 post-TBI at doses of 3 mg/kg (**B**) or 10 mg/kg (**C**). (**D**) Survival efficacy of LA-GM-CSF (3.0 mg/kg) in male mice given as a single or 2- or 3-dose regimen. (**E**) Survival efficacy in female mice by the administration of a single dose of LA-GM-CSF (3 mg/kg) 1-day post-TBI. Log-rank *p*-value of ≤0.0001 indicated as **** and *p* ≤ 0.05 as *, n = 25 animals/group.

A multi-dose regimen administered subcutaneously with the first dose prior to TBI was assessed for survival benefit at 10 mg/kg of LA-GM-CSF. The regimen of 3 days prior, 2 and 7 days post-administration (−3, +2, +7) resulted in 88% survival for the group administered 10 mg/kg LA-GM-CSF compared to 0% survival in the vehicle control group (Figure 2A). LA-GM-CSF, when administered post-TBI as a 3-dose regimen (on days 1, 6 and 11 post-TBI), showed maximum survival benefit at a lower dose of 3 mg/kg (79.2% survival at 7.9 Gy TBI, Figure 2B) compared to a higher dose of 10 mg/kg (20.8% survival at 8.1 Gy TBI, Figure 2C) in male mice. Statistical significance of the survival benefit against mice treated with vehicle (Synagis in PBS) was determined by the Log-rank test with a *p*-value of <0.0001 in the case of the 3 mg/kg group (Figure 2B) and *p* = 0.05 (Figure 2C). Survival efficacy of LA-GM-CSF was also tested as a 1-dose (day 1 post-TBI) and 2-dose (days 1 and 6) regimen, along with a repeat of a 3-dose regimen (days 1, 6 and 11) where each dose was at 3 mg/kg. The percentage survival in the LA-GM-CSF groups were 87.5%, 91.6% and 91.6%, respectively, while the age-matched cohort given vehicle on day 1 post-TBI showed 50% survival (Figure 2D). Survival benefit of the single dose of LA-GM-CSF at 3 mg/kg was confirmed in female mice at 8.25 Gy, as 64% compared to the vehicle group with 28% survival (Figure 2E, Log-rank *p* = 0.05).

### 2.3. Accelerated Hematopoietic Recovery by a Single Dose of LA-GM-CSF (3 mg/kg)

**Figure 3:**
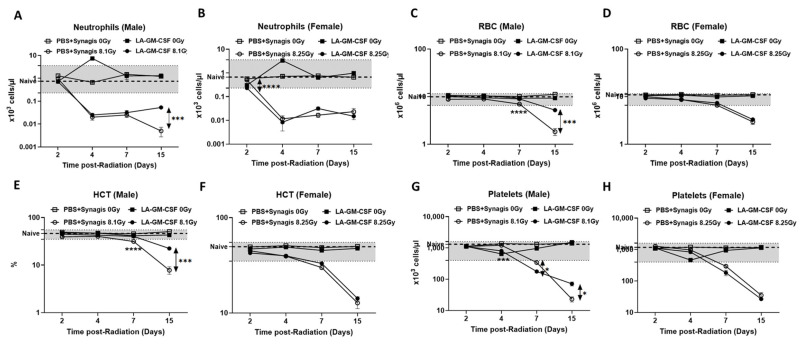
Complete blood cell counts in mice given LA-GM-CSF 24 h post-TBI as a single dose. Irradiated animals were given a single SC dose of either LA-GM-CSF (3 mg/kg) or vehicle (PBS + Synagis) 24 h post-TBI. Non-irradiated treatment groups were used as control and naïve (untreated, non-irradiated) mice were used as reference (dotted line) and the gray shaded area indicates the normal range in healthy mice based on the instrument’s manufacturer [28]. Complete blood counts were measured from the peripheral blood of mice on 2, 4, 7, and 15 days post-TBI exposure at 8.1 Gy (male mice) and 8.25 Gy (female mice). Data for neutrophils (**A**,**B**), red blood cells (RBC) (**C**,**D**), hematocrit (HCT) (**E**,**F**) and platelets (**G**,**H**) are plotted as mean ± SEM. Statistical significance was indicated as **** (*p* ≤ 0.0001), *** (*p* ≤ 0.001), and * (*p* = 0.015). Significant recovery in CBCs was observed in males with LA-GM-CSF administration when compared to the irradiated vehicle group. n = 6 animals/ group.

CBCs were monitored in the whole blood over 15 days post-TBI in male and female mice exposed to radiation (8.1 Gy and 8.25 Gy, respectively). The non-irradiated LA-GM-CSF group had similar cell counts as the non-irradiated vehicle control group or the naïve (no injection or irradiation) mice, except on day 4, slightly elevated levels of neutrophils were observed in both sexes, though within the normal range in females. In irradiated groups, the cell counts for neutrophils dropped significantly on day 4; however, in males, there was significant recovery (*p* < 0.001) on day 15 (0.05 ± 0.01 × 10^3^ cells/µL) in the LA-GM-CSF group compared to the vehicle-treated group (0.01 ± 0.01 × 10^3^ cells/µL) (Figure 3A). This recovery was not seen in the females (Figure 3B). In the case of RBC levels (Figure 3C), the irradiated LA-GM-CSF group had significantly higher (*p* ≤ 0.001) cell numbers on days 7 and 15 post-TBI (8.95 ± 0.06 × 10^6^ cells/µL and 5.19 ± 0.05 × 10^6^ cells/µL) compared to corresponding vehicle groups (6.96 ± 0.14 × 10^6^ cells/µL and 1.82 ± 0.31 × 10^6^ cells/µL respectively). There were no significant differences in RBC counts between the two irradiated female groups (Figure 3D). Effects on the hematocrit (HCT) were similar to those of RBC counts where in male mice, %HCT values (Figure 3E) were significantly higher (*p* < 0.001) in irradiated drug group on days 7 and 15 post-TBI (40.12 ± 0.23%, 22.32 ± 0.29%, respectively) compared to irradiated vehicle groups on the same days (31.33 ± 0.66%, 7.80 ± 1.40%, respectively). There were no significant differences in %HCT among irradiated female mice groups (Figure 3F). Though on day 7, the platelet count (Figure 3G) was higher in the vehicle-treated group (*p* = 0.015), there was significant recovery on day 15 (*p* = 0.015) in the LA-GM-CSF group (irradiated) with 71.33 ± 10.25 × 10^3^ cells/µL when compared to the vehicle group with 23.00 ± 4.30 × 10^3^ cells/µL. Interestingly, there was no significant difference in the platelet counts between the female irradiated groups (Figure 3H).

### 2.4. Detection of GM-CSF in Serum of Irradiated and Non-Irradiated Mice over Time

**Figure 4:**
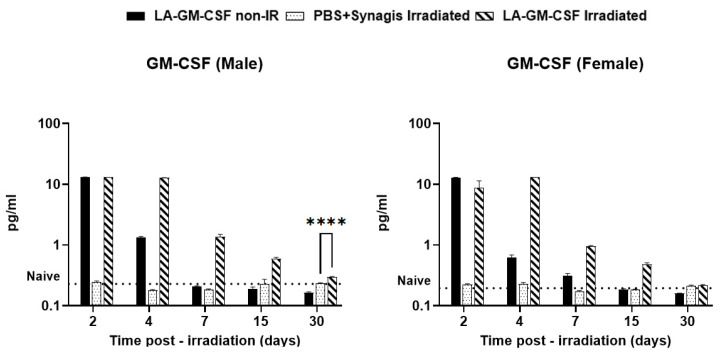
Detection of circulatory levels of GM-CSF until 30 days post-TBI. Circulatory levels of GM-CSF were detected using ELISA. Serum from non-irradiated and irradiated male and female mice was collected at various time points (days 2, 4, 7, 15, and 30 post-TBI) and analyzed for GM-CSF. In the groups where a single dose of 3 mg/kg LA-GM-CSF was administered on day 1 post-TBI (in irradiated mice), significantly high levels of GM-CSF were detected as expected. These levels declined over the 30 days in both irradiated and non-irradiated mice. In irradiated mice, GM-CSF levels stayed longer than in the non-irradiated group and also significantly higher than naïve levels (dotted line) until day 30 (in males **** *p* ≤ 0.0001).

Circulating levels of GM-CSF were quantified in serum for irradiated and non-irradiated male and female mice (Figure 4). The groups of mice received a single SC administration of LA-GM-CSF at 3 mg/kg body weight, 1-day post-irradiation. Serum was collected from all groups on days 2, 4, 7, 15 and 30 with respect to the day of irradiation (day 0). At the earliest time point (day 2), the amount of GM-CSF present in both the irradiated and non-irradiated samples was roughly the same, followed by a rapid decline in GM-CSF levels in the non-irradiated groups (males and females) whereas irradiated LA-GM-CSF groups maintained relatively higher levels of GM-CSF, even if their levels also decreased, the rate of decrease was slower, or the absolute levels remained higher (significantly higher (*p* ≤ 0.0001 on day 30 in males). Levels of GM-CSF in vehicle-treated irradiated groups (males and females) were similar to the levels in naïve animals (dotted line Figure 4).

### 2.5. LA-GM-CSF Given as a Single Dose of 3 mg/kg Post-TBI Improved Hematopoietic Recovery

**Figure 5:**
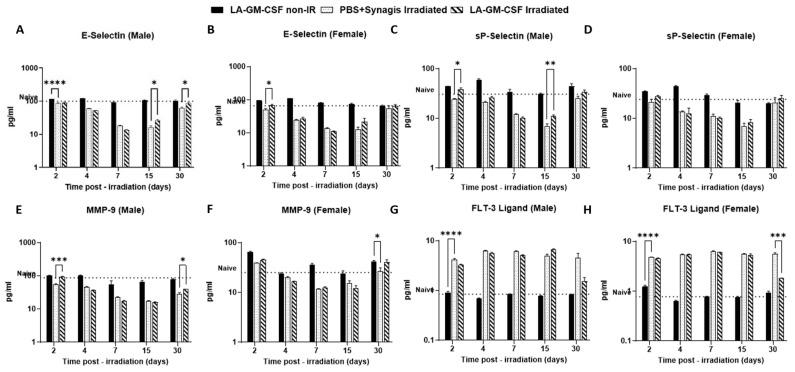
Differential expression of circulatory endothelial dysfunction markers in mice given LA-GM-CSF (3 mg/kg) 24 h post-TBI. Biomarkers of endothelial and vascular injury E-selectin (**A**,**B**), sP-Selectin (**C**,**D**), MMP-9 (**E**,**F**) and FLT-3 Ligand (**G**,**H**) were measured in serum collected from mice on days 2, 4, 7, 15 and 30 post-TBI by ELISA. Non-radiated drug group was used as a control and naïve values were used as a reference (dotted line). The data is represented as mean ± SEM. Statistical significance was determined by two-way ANOVA **** *p* ≤ 0.0001, *** *p* ≤ 0.001, ** *p* ≤ 0.01, * *p* ≤ 0.05).

To assess the effect of LA-GM-CSF on recovery of the immune system and vasculature in irradiated animals, we measured the biomarkers in serum collected on days 2, 4, 7, 15 and 30 post-TBI from male and female mice (Figure 5). The non-irradiated drug group was used as a control and naïve (untreated, non-irradiated) animals were used as a reference (dotted line).

As early as day 2 post-TBI, E-Selectin levels significantly dropped (*p* ≤ 0.0001) in the irradiated vehicle group compared to the non-irradiated group or naïve mice. Even though there was a gradual decline in the levels in the irradiated LA-GM-CSF group as well, the levels were higher (*p* ≤ 0.05) compared to the irradiated vehicle group on days 15 and 30 in male mice (Figure 5A). The pattern was slightly different in female mice (Figure 5B), where the recovery in E-selectin levels was simultaneous in the two irradiated groups.

**Figure 6:**
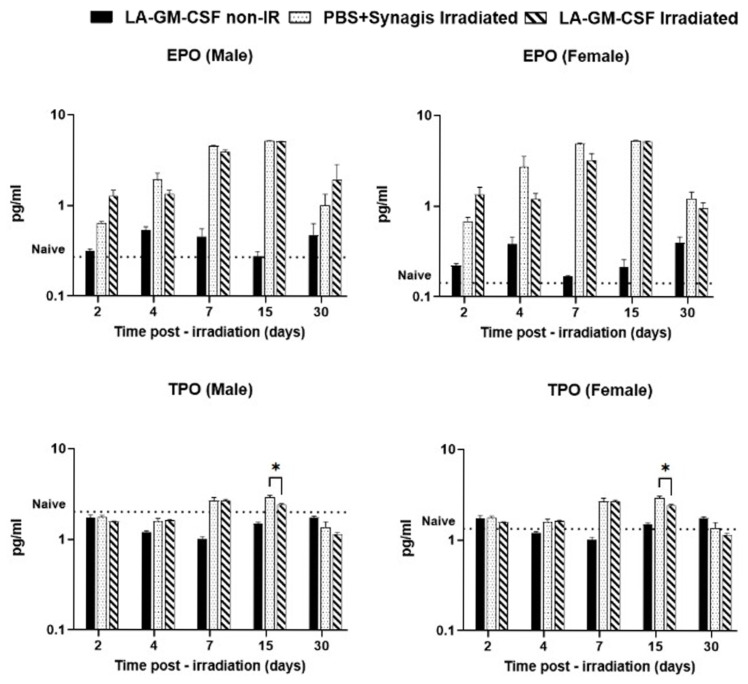
Differential expression of immune markers in mice given LA-GM-CSF (3 mg/kg) 24 h post-TBI. Serum samples collected from mice on days 2, 4, 7, 15, and 30 post-TBI were analyzed via ELISA to measure biomarkers of immune system injury. A non-irradiated, drug-treated group served as a control, with naïve values providing a reference (dotted line). While the overall effect of radiation on these biomarkers was evident, our analysis revealed no significant difference between the vehicle and LA-GM-CSF treatment groups. Statistical significance was indicated as * (*p* ≤ 0.05).

Similar trends were observed in the case of sP-selectin levels in male and female irradiated mice (Figure 5C,D, respectively), where in male mice the sP-selectin levels stayed significantly higher in LA-GM-CSF group than vehicle treated one (day 2 *p* ≤ 0.05, day 15 *p* < 0.001)In male and female irradiated mice, matrix metalloproteinase (MMP-9) levels were initially higher in LA-GM-CSF groups compared to vehicle on day 2, but then decreased by day 4 and eventually recovered on day 30 (Figure 5E,F). FLT-3 ligand levels were relatively higher in vehicle-treated animals compared to LA-GM-CSF-treated groups (Figure 5G,H). In the case of all four endothelial dysfunction biomarkers discussed above, the protein levels in the non-irradiated LA-GM-CSF-treated group were similar to those of untreated naïve mice. Biomarker levels for the immune system, erythropoietin (EPO) and thrombopoietin (TPO) were also assessed in the serum at various time points in male and female mice. Even though differential levels of the proteins were observed as radiation damage, there was no significant difference between the vehicle-treated and LA-GM-CSF-treated mice (Figure 6).

**Figure 7:**
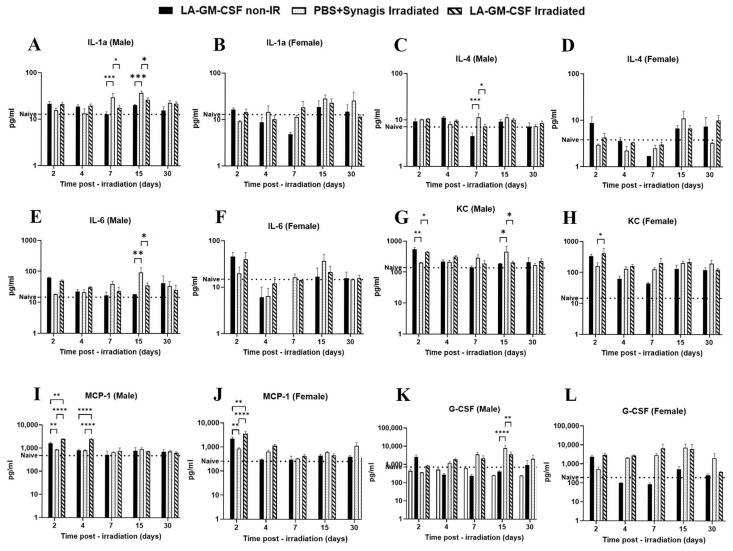
Differentially expressing cytokines, chemokines and growth factors in mice administered with LA-GM-CSF (3 mg/kg) 24 h post-TBI. Serum samples collected from mice at days 2, 4, 7, 15, and 30 post-TBI were analyzed with the Luminex assay to measure levels of 23 cytokines, chemokines and growth factors. A non-irradiated, drug-treated group (LA-GM-CSF non-IR) served as a control, with naïve values providing a reference (dotted line). Among the 23 biomarkers tested, six biomarkers (IL-1a (**A**,**B**), IL-4 (**C**,**D**), IL-6 (**E**,**F**), KC (**G**,**H**), MCP-1 (**I**,**J**) and G-CSF (**K**,**L**)) showed different levels. Most of these differences were found in male mice, with two specific biomarkers, MCP-1 and KC, also showing different levels in female mice. * *p* < 0.05, ** *p* = 0.008, *** *p* = 0.0002, and **** *p* < 0.0001.

Additionally, serum samples were also analyzed using a 23-plex cytokine panel. All comparisons were made with the non-irradiated LA-GM-CSF group, whereas untreated naïve animals were used as baseline (dotted line, Figure 7). Statistical analysis was done using two-way ANOVA and *p* < 0.05 is considered significant. Out of 23, 6 biomarkers displayed significant differential levels (Figure 7). Most changes were observed in males except for Monocyte chemoattractant protein-1 (MCP-1, Figure 7J) and Keratinocyte-derived chemokine (KC, Figure 7H). In male mice, Interleukins 1a, 4 and 6 (IL-1a, IL-4 and IL-6, respectively) showed significant increase in the irradiated vehicle group on either on day 7 (11.31 ± 2.33 pg/mL, *p* < 0.001, Figure 7C), day 15 (Figure 7E) or on both days (day 7: 29.70 ± 6.52 pg/mL, and day 15: 36.89 ± 3.75 pg/mL; *p* = 0.0002, Figure 7A). There was no significant difference in the levels of these 3 interleukins (Figure 7B,D,F) between the irradiated groups (vehicle and LA-GM-CSF) in female mice. In contrast, KC levels were seen to be significantly recovered in LA-GM-CSF groups on day 2 in males (*p* = 0.047) and females (*p* = 0.026) (Figure 7G,H). MCP-1 levels (Figure 7I,J) were higher on day 2 in non-irradiated LA-GM-CSF groups (1615.10 ± 182.91 pg/mL in males, 2200.28 ± 419.00 pg/mL in females) when compared to the respective naïve groups (479.00 ± 74.23 pg/mL in males, 254.6 ± 46.11 pg/mL in females), however irradiated vehicle groups had significantly (*p* < 0.0001) lower levels (857.24 ± 36.63 pg/mL in males and 855.79 ± 81.66 pg/mL in females). On day 4, MCP-1 levels stayed significantly higher (*p* < 0.0001) than the vehicle group in male mice (Figure 7I). In male mice, G-CSF levels in the LA-GM-CSF groups stayed higher than the vehicle group (not statistically significant) except on day 15, the vehicle group had 7926.72 ± 2935.62 pg/mL (*p* < 0.0001 versus non-irradiated group, *p* = 0.0066 versus irradiated LA-GM-CSF) as shown in Figure 7K.

GM-CSF levels in the non-irradiated and irradiated LA-GM-CSF-treated groups were significantly higher than the vehicle group on days 2 and 4 post-TBI and gradually declined to the normal levels (naïve group). Vehicle group had no change in GM-CSF levels compared to the naïve group at all time points.

### 2.6. LA-GM-CSF Accelerates Recovery of Sternal Bone Marrow Cellularity and Megakaryocytes

**Figure 8:**
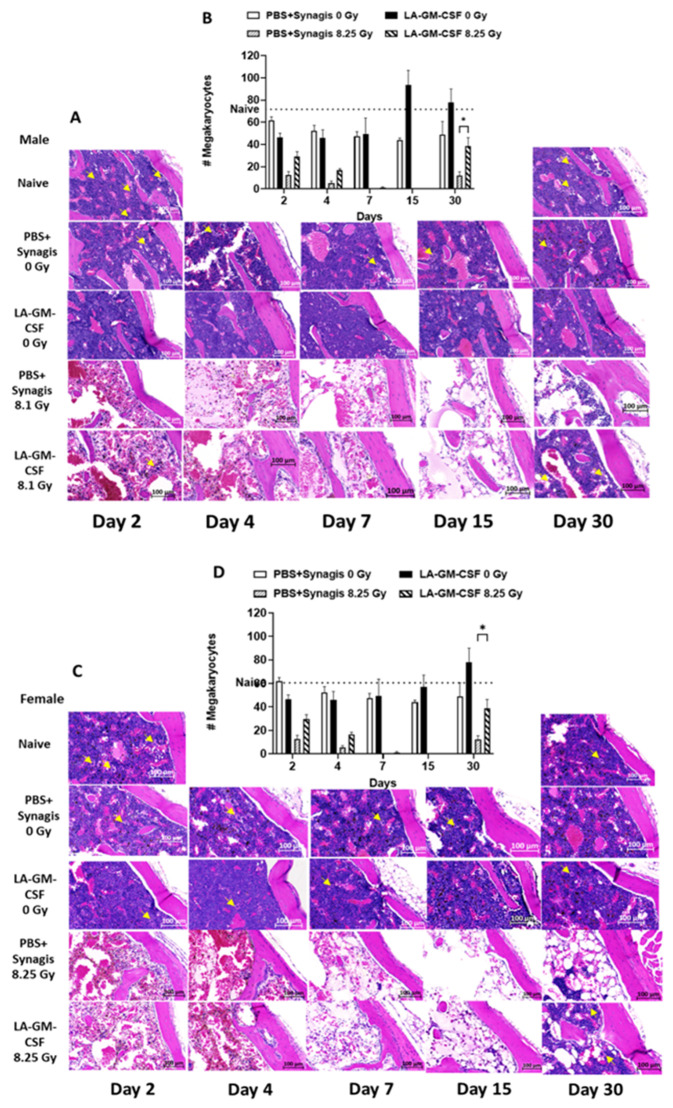
Sternal megakaryocyte recovery in mice given a single dose of LA-GM-CSF 24 h post-TBI. Representative H&E-stained sternal bone marrow sections for male mice. Megakaryocytes are identified by yellow arrows; the scale bar represents 100 μm (**A**) and the corresponding graph (**B**). Bottom panels show the H&E-stained section for the female mice and the corresponding megakaryocytes are identified by yellow arrows; the scale bar represents 100 μm (**C**) and the graphical representation of the data in (**D**). Irradiated or non-irradiated groups given the drug or vehicle were compared to each other, and naïve animals were used as reference (dotted line). The data is represented as mean ± SEM. Statistical significance was determined by two-way ANOVA * *p* ≤ 0.05.

Sterna were collected from male and female mice groups (Figure 8) and processed for histopathological evaluation. H&E-stained sections were evaluated for cellularity and megakaryocyte counts on days 2, 4, 7, 15 and 30. Our results show that in male mice (Figure 8A,B) LA-GM-CSF treatment increased megakaryocyte counts in radiated as well as non-irradiated groups compared to irradiated vehicle groups. However, in female mice (Figure 8C,D), non-radiated groups had significantly higher megakaryocyte counts for the LA-GM-CSF group compared to vehicle-treated cohorts. There was no significant difference between irradiated mice given LA-GM-CSF or vehicle treatment.

## 3. Discussion

Our studies investigated long-acting GM-CSF (LA-GM-CSF) as a countermeasure post-TBI in mice regarding its dose efficacy and timing of administration (Figure 2). Lower doses seem to have improved survival upon TBI exposure, with a 3 mg/kg multi-dose regimen showing higher survival compared to 10 mg/kg in male mice. The difference in efficacy, whereby the lower dose of 3 mg/kg is more efficacious than the higher dose, is in line with the known effects of GM-CSF in cancer immunotherapy [29]. In fact, higher doses of GM-CSF have been shown to induce immunosuppression via recruitment of myeloid suppressor cells [30]. This modulation of the immune response likely correlates with the outcomes observed for the differing dosing levels from a pharmacokinetic pharmacodynamic (PK/PD) standpoint; however, it is also possible that mechanisms such as receptor saturation could play a role in this difference. The consistently high survival rates observed with single, two, or three early doses of LA-GM-CSF indicate a possible regimen with early administration after radiation exposure (24 h post-TBI).

Our studies further showed that pre-TBI administration with LA-GM-CSF also conferred significant protection against lethal radiation exposure in mice with a dependence on timing of the dosing regimen for maximal survival benefit (Figure 3). Starting the multi-dose LA-GM-CSF regimen 72 h pre-TBI resulted in markedly higher survival rates compared to initiating the same regimen 24 h prior, for both 10 mg/kg and 3 mg/kg doses. In contrast, animals receiving vehicle experienced substantial mortality regardless of the pre-TBI start time, indicating the survival benefit due to LA-GM-CSF alone.

It is understood that ionizing radiation affects male and female species differently because of their differential radiosensitivity [31]. To evaluate hematological recovery post-TBI in male and female mice, we studied the effect of LA-GM-CSF administration post-TBI (Figure 4 and Figure 5). In male mice, post-TBI administration of LA-GM-CSF was associated with a significant recovery in red blood cell counts and hematocrit levels, particularly at later time points post-irradiation. It has been well known that different forms of GM-CSF have been used for the treatment of neutropenia and megakaryocyte stimulation [32,33]. Consistent with this, in our studies with LA-GM-CSF, neutrophil counts were elevated in treated males, and platelet counts exhibited an interesting pattern of initial suppression followed by subsequent recovery. In contrast, female mice displayed a less pronounced hematological response to post-TBI LA-GM-CSF treatment. Microscopic examination of sternal bone marrow samples (Figure 7) from male mice demonstrated that LA-GM-CSF administration led to an increase in megakaryocyte numbers in both irradiated and non-irradiated animals when compared to irradiated males receiving only the vehicle. In contrast, while non-irradiated females treated with LA-GM-CSF exhibited significantly higher megakaryocyte counts than non-irradiated females receiving the vehicle, there was no significant difference in megakaryocyte counts between irradiated female mice treated with LA-GM-CSF and those receiving the vehicle. These sex-specific hematological responses were further corroborated by our serum analysis of circulating injury biomarkers, including GM-CSF, e-selectin, sP-selectin, FLT-3 ligand, and MMP-9, which exhibited different time profiles and divergent patterns between male and female mice following LA-GM-CSF administration. These biomarkers were selected because of the multitude of damage caused by radiation exposure, including declines in bone marrow and blood cell lineages. It has been previously established that H-ARS is often accompanied by vascular dysfunction [34,35,36].

These appreciable differences in the response in male and female mice are noteworthy and warrant further consideration. Among potential reasons for these differences are PK/PD, given that we observed an effect in male mice that a lower dose resulted in a more efficacious outcome than a higher dose, it is similarly possible that further dose refinement could impact the survival and overall impact in female mice. While this should be explored in future studies, it must also be weighed against the regulatory pathway for ARS countermeasures, the FDA’s Animal Rule. From this perspective, the efficacy would need to be demonstrated in additional animal models and these findings should be kept in mind to determine if these differences are conserved across species. This may ultimately result in the need for differential dosing if further evidence supports it. Our results on LA-GM-CSF safety in male mice revealed some dose-related physiological changes and a general favorable safety margin towards lower doses (Figure 1). Both 15 mg/kg and 30 mg/kg doses appeared safe overall, but the 30 mg/kg dose caused a notable weight decrease on day 7. Serum chemistry analysis on day 25 showed differences with the 30 mg/kg dose, including lower BUN and ALT, and higher ALKP and AST levels, suggesting an influence on metabolic and liver functions. Hematological assessments indicated that both doses led to early increases in WBC and neutrophils, suggesting an inflammatory response. Subsequent changes in lymphocytes and dose-specific effects on monocytes and platelets point to a complex modulation of the immune system. Previous studies [37,38] have implicated the direct targets of GM-CSF, including matrix metalloproteins, and anti-apoptotic proteins (Mcl-1 and Bcl-2), as well as those markers which are indirectly or secondarily impacted by GM-CSF [39], including Interleukin 6 and 1β. However, the noted differences in PK/PD can alter the impacts on numerous pathways through differential dosing, so further studies would help to elucidate the role and exact mechanism of protection for LA-GM-CSF. These findings highlight the importance of careful dose selection in future applications to manage potential side effects while achieving the desired biological outcome.

## 4. Materials and Methods

### 4.1. Animals

Mice (C57BL/6, males and females, 11–14 weeks) were procured from Jackson Laboratory (Bar Harbor, ME, USA) and housed in a climate-controlled facility at the Department of Laboratory Animal Resources (DLAR) of the Uniformed Services University of the Health Sciences (USUHS). The USUHS DLAR facility is accredited by the Association for Assessment and Accreditation of Laboratory Animal Care International (AAALAC). All murine housing rooms are equipped with an automated 12/12 h light/dark schedule, and all animals were fed Harlan Teklad rodent diet and acidified water (pH 2.5–3.0) ad libitum. The USUHS Institutional Animal Care and Use Committee (IACUC) approved all protocols and animal procedures presented in this manuscript [40].

### 4.2. Ethics Statement

All experiments and studies were ethically conducted in accordance with protocols approved by the USUHS IACUC, strictly adhering to the guidelines set by the National Research Council’s Guide for the Care and Use of Laboratory Animals.

### 4.3. Total Body Irradiation (TBI) and Dosimetry

Prior to irradiation, animals were transported via climate-controlled van to the Armed Forces Radiobiology Research Institute (AFRRI) irradiation facility from the USUHS DLAR housing facility. For this transport, the individually ventilated cages used to house the animals in the DLAR facility were placed in secondary containers to reduce exposure to potential pathogens outside of the animal designated spaces. The Transport time was 10 min or less and a 60 min rest period was employed after animal transport prior to the start of irradiation procedures. Following irradiation, mice were returned to their home cages and transported back to the USUHS DLAR animal housing facility in the same climate-controlled van.

Simultaneous bilateral irradiations were employed in the AFRRI Cobalt-60 facility. For these exposures, mice were placed in custom Lucite boxes (up to 8 animals in each box) arranged in a 5 × 6 grid-like pattern. Prior to irradiation, dose rates were measured in the core of acrylic phantoms (dimensions 3” long × 1” diameter) placed in the grid array used for animal irradiation utilizing an alanine/Electron Spin Resonance (ESR) dosimetry system (American Society for Testing and Material Standard E 1607) [41]. The calibration curve was constructed based on dosimeters with traceability to the National Institute of Standard and Technology (NIST, Gaithersburg, MD, USA), with verification via inter-comparison with the National Physical Laboratory (NPL) in the United Kingdom, as reported previously [42,43]. During irradiation, animals were restrained in the Lucite boxes for the minimum time required to complete the irradiation; this time did not exceed 30 min. Following irradiation, animals were returned to their home cages with ad libitum access to food and water and ultimately transported back to their assigned housing room.

### 4.4. Veterinary Care Following Radiation

Following irradiation, animals were monitored up to four times daily. Irradiated animals experience a critical period during which signs and symptoms of pain and distress are common. This period is typically 14 continuous days beginning on the ~12th day of the study and continuing through the 26th day of the study for C57BL/6 mice. Deceased animals found in the course of the study were removed during the 4× daily checks and documented in the study record. Predetermined criteria were used to assess pain and distress, refined from parameters established previously by Koch et al. [44]. These criteria involve scoring of unresponsiveness, abnormal posture, unkempt appearance, immobility, and lack of coordination. Moribund mice were humanely euthanized based on scores exceeding 12 from the pain and distress criteria mentioned above. Mice that were unable to stand upright were immediately euthanized according to American Veterinary Medical Association (AVMA) guidelines.

### 4.5. LA-GM-CSF

LA-GM-CSF was manufactured and supplied by the California Institute for Biomedical Research (Calibr), a division of Scripps Research (La Jolla, CA, USA), as described previously [21]. LA-GM-CSF was supplied as a solution in PBS containing Synagis (vehicle). It was stored at −80 °C until use. Prior to dosing, it was diluted to the appropriate concentration in PBS and administered to mice as a subcutaneous (SC) injection on the nape of the neck of 0.1 mL volume.

### 4.6. Fourteen-Day Acute Safety Studies

C57BL/6 male mice (n = 5/group) were weighed and distributed into two groups (vehicle control and LA-GM-CSF). An untreated control (naïve control) was used to account for the average growth of animals in the batch. Animals were injected SC at the nape of the neck 15 mg/kg or 30 mg/kg of LA-GM-CSF as a multidose regimen starting on day 1, followed by days 6 and 11 or PBS containing Synagis (vehicle) in a volume of 0.1 mL.

Animals were monitored for acute signs of toxicity on days of dosing, continuously for the first hour, with a secondary check 4 h after the dose was administered, followed by daily checks for 14 days following the final dose [45]. During the study, defined intervals (0 (1 h prior to dosing), 3, 7, 9, 15, 18, and 25 d) were employed for body weight and blood collection for counting complete blood cells (CBCs). Blood (20 µL) was collected via the submandibular vein for hematological analysis. At the conclusion of the safety study, animals were anesthetized with isoflurane and blood collected via cardiac stick, followed by human euthanasia via cervical dislocation. Following euthanasia, gross anatomy was investigated for abnormalities in major organs at necropsy. Following cardiac stick, blood was separated into serum (BD Microtainer tubes, 36596, Fisher Scientific, Pittsburgh, PA, USA) via centrifugation (2400× *g*, 10 min) for hepatic and renal panel chemistry evaluations [40].

### 4.7. Determine Thirty-Day Survival Efficacy of LA-GM-CSF as Prophylactic as Well as Mitigator

Irradiation survival studies fall under the guidelines of the FDA animal rule, as testing in humans is not possible [46]. Based on previous approvals from the FDA, murine models are acceptable for the development of radiation countermeasures; therefore, we used this model to evaluate the efficacy of LA-GM-CSF.

### 4.8. Radioprotective Efficacy of LA-GM-CSF with Multiple Dose Regimen

To investigate the radioprotective efficacy of LA-GM-CSF, baseline body weights were collected prior to irradiation, animals weighing ±10% of the mean weight were removed, and the remaining animals were randomly assigned to one of two groups (vehicle control and LA-GM-CSF). Mice were administered either 3 or 10 mg/kg LA-GM-CSF starting at 24 or 72 h prior to radiation as 1st dose and 2nd and 3rd dose at day 6 and day 11 following the first dose by SC injection at the nape of the neck in 0.1 mL using a 23-G needle. PBS containing Synagis was used as a vehicle control in a volume of 0.1 mL. The treatment groups for LA-GM-CSF and its vehicle comprised 25 animals and the dose of radiation was 7.9 to 8.1 Gy. At the end of the study, Kaplan–Meier survival curves were plotted, and statistical significance was determined by log-rank and Fisher’s exact analysis. All statistical analyses were completed using GraphPad Prism 10 software [40].

### 4.9. Radiomitigative Efficacy of LA-GM-CSF with Single or Multiple Dose Regimen

To investigate the radiomitigative efficacy of LA-GM-CSF, mice were randomly assigned to two groups (vehicle control and LA-GM-CSF) with five animals per cage. Mice were administered either 3 or 10 mg/kg LA-GM-CSF starting at 24 h post-radiation as the 1st dose and the 2nd and 3rd doses at day 6 and day 11 following the first dose by SC injection at the nape of the neck in 0.1 mL using a 23-G needle. PBS containing Synagis was used as a vehicle control in a volume of 0.1 mL. Each treatment group for the drug and its vehicle contained 25 animals, and the radiation dose for males was 7.9 to 8.1 Gy and 8.25 Gy for females. These doses correspond to a dose that produces 70% lethality in 30 days and takes into account the difference in radiosensitivity observed between the sexes [47]. Survival was monitored up to four times a day for 30 days and surviving animals were euthanized at the completion of the study. Survival data were plotted as Kaplan–Meier plots and the statistical significance of the survival differences was determined by log-rank and Fisher’s exact tests using GraphPad Prism 10 software [47].

### 4.10. Hematopoietic Recovery with LA-GM-CSF Administered a Single Dose of 3 mg/kg 24 h Post-TBI in Males and Females

To study the mitigative effects of LA-GM-CSF on the recovery from hematopoietic injury following TBI, mice (n = 6/group) were treated with either a single dose of the test article (3 mg/kg) or its vehicle 24 h post-TBI at a radiation dose of 8.1 Gy for males and 8.25 Gy for females. In addition, groups administered LA-GM-CSF or its vehicle in the absence of radiation were utilized. Terminal Blood collection was performed on days 2, 4, 7, and 14 post-TBI under isoflurane anesthesia using a 23 G needle via cardiac stick, followed by humane euthanasia as per IACUC protocol. CBC/differential analysis was conducted on ~20 µL of blood, which was collected into EDTA-coated tubes and was continually rotated from collection until analysis. CBC data was measured on a HESKA Element HT™ 5 Analyzer system (HESKA Corporation, Loveland, CO, USA). Data from this analysis included WBC, NEU, MON, LYM, RBC, HCT, and PLT counts. The rest of the blood was separated for serum and used to quantify levels of circulatory proteins via Enzyme-Linked ImmunoSorbent Assay (ELISA) and estimate inflammatory cytokines and growth factors.

Following blood collection, animals were euthanized and sternal bone marrow was harvested for histopathology and femoral bone marrow for clonogenic assay [42,43].

### 4.11. Sternum Bone Marrow Histopathology

Freshly collected sterna were fixed in 10% neutral-buffered formalin for a minimum of 24 h, then decalcified in 12–18% sodium EDTA (pH 7.4–7.5). Following decalcification, a series of graded ethanol solutions was used; the samples were embedded in paraffin and sectioned at 5 µm thickness. These sections were rehydrated and stained with hematoxylin and eosin (H&E), prior to mounting on microscopy slides. Whole-section imaging was performed using a Zeiss Axio 7 scanner (Zeiss Microscopy, LLC, White Plains, NY, USA), and megakaryocytes were quantified across the entire section for each animal [42,48].

### 4.12. Hematopoietic Progenitor Clonogenic Assay

Using a 23G needle, freshly harvested femoral bones were flushed with Iscove’s modified Dulbecco’s medium, and collected cells were processed and cultured (Mouse Colony-Forming Cell Assays Using MethoCult, Stem Cell Technologies, Cambridge, MA, USA). Following a 14-day incubation period at 37 °C (with 5% CO_2)_, granulocyte–macrophage colony-forming units (CFU-GM), granulocyte-erythrocyte-monocyte-macrophage CFU (CFU-GEMM), colony-forming unit-erythroid (CFU-E) and erythroid burst-forming units (BFU-E) were quantified using STEMvision™ colony counter (Stem Cell Technologies, Vancouver, BC, Canada) [42,48].

### 4.13. Serum Levels of GM-CSF, E-Selectin, sP-Selectin, Flt3-Ligand, MMP-9, EPO and TPO

Serum was separated from the blood collected at different time points. Circulatory levels of mouse GM-CSF, E-selectin, Flt3 ligand, MMP-9, EPO and TPO were measured using Quantikine ELISA kits purchased from R&D systems (McKinley, MN, USA) using the standard curve following the manufacturer’s instructions [42,43]. The cytokine detection limits were 18 pg/mL, >20 pg/mL and >5 pg/mL for TPO, EPO and Flt3L ELISAs, respectively [34].

### 4.14. Estimation of Inflammatory Cytokines and Growth Factors in Serum

Serum samples from hematopoietic recovery studies were analyzed via the Bio-Plex Pro Mouse Cytokine 23-plex assay kit (BioRad, Hercules, CA, USA) following the manufacturer’s instructions [48]. Briefly, 50 µL of standards or samples were used with 50 µL of magnetic beads provided with the kit. The following cytokines, chemokines, and growth factors were assessed: Eotaxin, G-CSF, GM-CSF, IFN-γ, IL-1α, IL-1β, IL-2, IL-3, IL-4, IL-5, IL-6, IL-9, IL-10, IL-12 (P40), IL-12 (P70), IL-13, IL-17A, KC, MCP-1, MIP-1α, MIP-1β, RANTES, and TNF-α. Washing steps were completed as instructed using a Bio-Plex Pro washer following the manufacturer’s protocol. Biotinylated detection antibody cocktail and streptavidin-phycoerythrin were quantified via the vendor’s protocol. A Bioplex 200 (BioRad, Hercules, CA, USA) was utilized to read the plate, and the data was acquired via the Bio-Plex Manager 6.1.1 [48].

### 4.15. Statistical Analysis

Kaplan–Meier plots were used to assess survival studies. From these plots, Fisher’s exact test at 30 days was used to assess survival, and log-rank analysis was used to determine statistically significant differences between individual survival curves. Both analyses were conducted utilizing GraphPad Prism 10 software. For all statistical tests, *p*-values less than 0.05 were considered significant. For analyses conducted that did not consider animal survival, data were plotted as means and standard errors. For comparison between three or more groups, analysis of variance (ANOVA) is used to determine significant differences among the different groups. For comparisons between two groups, significant differences were identified using an unpaired *t*-test.

## 5. Conclusions

Collectively, our results underscore the significant potential of LA-GM-CSF as a therapeutic and prophylactic agent against radiation-induced injury. The results showing administration timing and sex-specific differences in hematological recovery and bone marrow stimulation necessitate further investigation to optimize intervention. While lower doses appear efficacious and generally well-tolerated, a thorough understanding of the potential dose-related effects of LA-GM-CSF on metabolic parameters is essential for effective management of the potential consequences of radiation exposure.

## Data Availability

Further information and requests for resources and reagents should be directed to Sanchita P. Ghosh (sanchita.ghosh@usuhs.edu). Materials availability: This study did not generate new reagents. Data availability: The datasets generated during and/or analyzed during the current study are available from the corresponding author on reasonable request. Any additional information required to reanalyze the data reported in this paper is available from the lead contact upon request.

## Abbreviations

The following abbreviations are used in this manuscript:

%	Percent
°C	Celsius
µL	Microliter
µm	Micrometer
AAALAC	Assessment and accreditation of laboratory animal care international
AFRRI	Armed forces radiobiology institute
ALKP	Alkaline phosphatase
ALT	Alanine aminotransferase
ANC	Absolute neutrophil counts
ANOVA	Analysis of variance
ARS	Acute radiation syndrome
AST	Aspartate aminotransferase
AVMA	American Veterinary Medical Association
BFU-E	Erythroid burst-forming units
BUN	Blood urea nitrogen
Calibr	California institute of biomedical research
CBC	Complete blood count
CFU	Colony-forming units
CFU-E	Colony-forming unit erythroid
CNS-ARS	Central nervous system acute radiation syndrome
CO_2_	Carbon dioxide
dL	deciliter
DLAR	Department of laboratory animal resources
EDTA	Ethylene diamine tetraacetic acid
ELISA	Enzyme linked immune sorbent assay
EPO	Erythropoietin
ESR	Electron spin resonance
FDA	Food and drug administration
Flt3l	FMS-like tyrosine 3 ligand
g	gram
G-CSF	Granulocyte colony-stimulating factor
GEMM	Granulocyte erythrocyte monocyte-macrophage
GI-ARS	Gastro intestinal acute radiation syndrome
GM-CSF	granulocyte–macrophage colony-stimulating factor
Gy	Gray
h	Hour/hours
H&E	Hematoxyline and Eosin
H-ARS	Hematopoietic acute radiation syndrome
HCT	Hematocrit
IACUC	Institutional animal care and use committee
IFN-γ	Interferon- gamma
IL	Interleukin
KC	Keratinocyte chemoattractant
LA-GM-CSF	Long-acting granulocyte–macrophage colony-stimulating factor
LYM	Lymphocytes
MCP-1	Monocyte chemoattractant protein-1
mg/kg	Milligram/kilogram
MIP	Macrophage inflammatory protein
mL	milliliter
MMP-9	Matrix metallo proteinase -9
MON	Monocytes
NIST	National institute of standard and technology
NPL	National physics laboratory
PBS	Phosphate-buffered saline
PK/PD	Pharmacokinetic Pharmacodynamic
pg	Picogram
PLT	Platelets
RANTES	Regulated upon activation, normal T cell expressed and secreted
RBC	Red blood cells
SC	Subcutaneous
SEM	Standard error mean
TBI	Total body irradiation
TNF	Tissue necrosis factor
TPO	Thrombopoietin
TPOm	Thrombopoietin mimetic
USUHS	Uniformed services university of the health sciences
WBC	White blood cells
